# Low-Dose Mitomycin C Decreases the Postoperative Recurrence Rate of Pterygium by Perturbing NLRP3 Inflammatory Signalling Pathway and Suppressing the Expression of Inflammatory Factors

**DOI:** 10.1155/2019/9472782

**Published:** 2019-11-15

**Authors:** Qie Guo, Xiao Li, Meng-Na Cui, Yu Liang, Xiang-Peng Li, Jun Zhao, Li-Na Wei, Xiao-Lei Zhang, Xiang Hua Quan

**Affiliations:** Department of Clinical Pharmacy, The Affiliated Hospital of Qingdao University, Qingdao, Shandong 266003, China

## Abstract

A pterygium is generally believed to be a chronic inflammatory lesion caused by external stimuli that develops from the conjunctiva and grows onto the cornea. Simple bare sclera excision is the most commonly used method to treat pterygium. However, the high postoperative recurrence rate of pterygium remains a persistent challenge. Mitomycin C (MMC) is an antineoplastic antibiotic that inhibits DNA, RNA, and protein synthesis. In recent years, although MMC has proven useful for the treatment of pterygium, its application has been controversial because of its clear toxicity and the possibility of ocular complications. In the current study, we prospectively recruited patients to receive or not receive a local injection of MMC (0.4 mg/ml). Follow-up was conducted with the patients to determine the postoperative recurrence rate of pterygium and/or to observe any ocular complications. The remarkable results demonstrated that MMC can decrease the postoperative recurrence rate of pterygium without leading to serious eye complications. Further results indicated that MMC can inhibit the activation of the NLRP3 inflammatory signalling pathway and thus downregulate the expression of downstream molecules, including IL-18 and IL-1*β*. MMC also reduced the expression of inflammatory factors TGF-*β*1, VEGF, and IL-6. In addition to influencing these factors, MMC suppressed neovascularization and the proliferation of corneal fibroblasts to effectively reduce the recurrence rate of pterygium. Taken together, our results provide a theoretical basis for the development of prevention and treatment strategies for pterygium and suggest that MMC is highly effective as an adjunctive treatment after excision of primary pterygia.

## 1. Introduction

A pterygium is a fibrovascular and wing-shaped neoplasm that can invade the cornea and cover the pupil area [1]. Pterygia are common causes of induced astigmatism, ocular movement restriction, and visual impairment [2]. There are many treatments for pterygium. Antibiotic eye drops can be used to relieve congestion and control conjunctivitis. Corticosteroid eye drops can also be used when severe congestion occurs [3]. Cryotherapy is used in the treatment of relatively small and thin pterygia to destroy and atrophy new blood vessels [4]. However, surgical excision is the main treatment for pterygium, although the recurrence rates after simple excision are as high as 30.0%∼88.9% [5]. Revealing the mechanism of pterygium recurrence and how to reduce the recurrence rate has become the topics of considerable focus.

Accumulating evidence indicates that infiltration of inflammatory cells, active fibroblasts, and abundant capillaries is found in pterygium specimens [6]. Moreover, inflammatory factors produced by inflammatory cells or released through paracrine secretion can induce fibrosis and neovascularization, which are key factors for the occurrence and development of pterygium [7]. TGF-*β*1 and VEGF induce pterygium production by increasing vascular permeability and promoting angiogenesis and fibroblast proliferation in the conjunctival mucosa [8]. In particular, activation of the TGF-*β*1/smad6 or TGF-*β*1/smad7 pathway can promote fibroblast proliferation and the degradation of the extracellular matrix of conjunctival fibroblasts by inducing IL-6 gene transcription, leading to the occurrence of pterygium [9]. Aberrant expression of IL-8 in pterygium epithelial cells, vascular endothelial cells, and the basement membrane has also been shown to be associated with angiogenesis and fibroblast proliferation during the formation of pterygium [10]. Nevertheless, whether high expression of TGF-*β*1, VEGF, IL-6, and IL-8 can also promote the recurrence of pterygium is seldom reported.

Inflammasomes are intracellular protein complexes that are composed of apoptosis-associated particulate proteins, caspases, and NOD-like receptors and are stimulated by pathogen-associated molecular patterns (PAMPs) or danger-associated molecular patterns (DAMPs) [11]. Inflammasomes play a key role in innate immune defence by phosphorylating and cleaving Caspase-1 to promote the maturation of the cytokine precursors pro-IL-1 and pro-IL-18, by which diverse downstream signalling pathways and proinflammatory processes are initiated, including Caspase-1-dependent programmed cell death [12]. The most well-characterized inflammasome subtype, the NOD-like receptor family pyrin domain-containing 3 (NLRP3) inflammasome, has been more heavily focused on than other subtypes because its activation is thought to be involved in the immune responses of many diseases, such as asthma [13] and diabetes [14]. However, limited information is available on the association between the NLRP3 inflammasome and pterygium development.

Mitomycin C (MMC) is an antitumour antibiotic that promotes DNA depolymerization and inhibits RNA-dependent DNA synthesis, thereby inhibiting tumour cell proliferation [15]. In recent years, MMC has also been considered as an adjuvant therapy for pterygium; however, there is no uniform conclusion about the administration route, dosage, or even the underlying mechanism of MMC in the treatment of pterygium.

Herein, to identify a novel agent that reduce recurrent pterygium and supply a promising strategy for pterygium treatment, a single dose of MMC (0.4 mg/ml) was locally injected into the pterygium tissues of patients with primary pterygium before bare sclera excision. The results from clinical follow-up observations showed that preoperative injection of a low dose of MMC can reduce the postoperative recurrence rate of pterygium without causing serious eye complications. Further results demonstrated that MMC injection can significantly inhibit the activation of the NLRP3/Caspase-1 pathway in pterygium tissues and thus decrease the expression of IL-1*β* and IL-18. Moreover, reduced expression of TGF-*β*1, VEGF, and IL-6 in pterygium tissues was also confirmed after MMC administration. Most importantly, these mechanisms work together to inhibit angiogenesis and fibroblast proliferation, which has been deemed responsible for pterygium recurrence.

Taken together, these results suggest that MMC not only inhibits tumour proliferation but also suppresses the activation of NLRP3 pathways and decreases the expression of inflammatory factors to prevent fibroblast proliferation and angiogenesis, thereby reducing the recurrence rate of pterygium.

## 2. Materials and Methods

### 2.1. Subjects

Ninety patients with primary pterygium, including 48 males and 42 females ranging in age from 32 to 51 years, were enrolled. All of the patients were treated at the Affiliated Hospital of Qingdao University between February 2013 and June 2014 and were characterized as having fibroblastic proliferation of conjunctival or subconjunctival tissues and triangular invasion of the cornea of more than 2 mm. None of the patients had other ocular surface diseases or a history of drug use. All patients underwent pterygium excision by the same surgeon and were randomly divided into two groups according to whether they received MMC before surgery (group I, *n* = 45, received MMC injection; group II, *n* = 45, did not receive any drug treatment). Pterygium samples were obtained from 45 patients (45 eyes) in group I and from 45 patients (50 eyes) in group II. Normal conjunctival tissue samples from five cases of sudden death were provided by the eye bank of Qingdao Ophthalmological Hospital.

All tissue specimens were preserved in liquid nitrogen for western blotting analysis, fixed with 4% paraformaldehyde for haematoxylin and eosin (HE) staining, and paraffin-embedded for immunohistochemical analysis.

This study was approved by the ethics committee of the Affiliated Hospital of Qingdao University, and *the Research ethics committee license number* is QYFYKYLL-2013-04-28-02. Informed written consent was obtained from all patients.

### 2.2. MMC Treatment

Tetracaine (1%) was used for topical anaesthesia before injection, and then MMC (0.4 mg/ml) was injected directly into the central part of the head or body of the pterygium once every three days before pterygium excision. The best effect was achieved when apophysis was observed.

### 2.3. Evaluation of the Effect of Pterygium Excision

The following indicators were used to classify an operation as successful:

1. The head of the pterygium was paler and the edge of the head more membranous than before; in addition, the blood vessels were thinner or had disappeared
2. The body of the pterygium was congested, and the blood vessels were atrophied to varying degrees
3. The head was adhered to the cornea, and this adhesion was easy to peel off

### 2.4. Follow-Up Visits to Assess Ocular Complications

Serious complications from local injection of MMC in the indicated patients were assessed according to the appearance of noticeable symptoms such as eye pain and a foreign-body sensation and ocular surface observation of congestion, oedema, ulceration, corneal colouration, and lens opacity.

Follow-up visits were scheduled for postoperative days 1, 3, 7, 15, and 30 and every one to two months thereafter. The whole follow-up period lasted one year.

### 2.5. Observation of Postoperative Recurrence

From the second month after excision, follow-up visits were executed once a month for two years, and the following grading method was used to determine whether the pterygium was recurrent:

  Grade I: the operative site showed a normal appearance of the bulbar conjunctiva
  Grade II: the neovascularization extended to the cornea, but there was no hyperplasia of fibrous tissue
  Grade III: fibrous tissue proliferated significantly but did not invade the cornea
  Grade IV: there was a substantial recurrence of pterygium

### 2.6. Reagents and Antibodies

MMC was obtained from R&D Systems, Inc. (Minneapolis, USA). Rabbit antihuman NLRP3(#13158), rabbit antihuman IL-1*β*(#12703), rabbit Caspase-1(#3866), rabbit antihuman Cleaved Caspase-1(#4199), rabbit antihuman TGF-*β*1(#5154), rabbit antihuman VEGF(#2445), and rabbit antihuman IL-6(#12153), rabbit antihuman IL-8(#94853), rabbit antihuman TNF-*α*(#8184) monoclonal antibodies (mAbs), and horseradish peroxidase (HRP)-conjugated secondary(#7075) mAbs were purchased from Cell Signaling Technology.

### 2.7. Western Blot Analysis

Total protein from cell or tissue specimens was obtained using RIPA Lysis Buffer (Solarbio Science & Technology Co., Ltd., Beijing, China). Equal amounts of protein samples were separated by 12% SDS-PAGE gels and transferred to the nitrocellulose membranes (Millipore, Bedford, USA). The membranes were blocked in Tris-buffered saline with 5% (w/v) nonfat dry milk and then incubated with primary antibodies overnight at 4°C, followed by incubation with goat anti-rabbit IgG H&L (HRP) (1 : 100 dilution, ab6721, Abcam) for 1 h at room temperature. Immunoreactive proteins were visualized by the ChemiDoc ™ XRS + System (Bio-Rad) Immuno-reactive proteins were visualized using immobilon western chemiluminescent HRP substrate (Millipore). Relative intensities of indicated proteins towards *β*-actin were also evaluated.

### 2.8. Quantitative Real-Time PCR

Total RNA from indicated cells was extracted by the TRizol regent (Invitrogen), and cDNAs were synthesized using SuperScript™ III (Thermo Scientific), followed by using the real-time PCR analysis with the assistance of TaqMan™ Master Mixes (Thermo Scientific) towards the expression of IL-18, according to the manufacturer's procedure. GAPDH was used as the internal reference. The primer sequences of IL-18 and GAPDH genes are as follows: IL-18 (Forward:5′-GCCAAAGCTTATGACCATGAGACACACAACTG-3′, Reverse: 5′-GGAATTCGACTTAACCCTGCTGCTGTGTGGACT-3′5′-TACCA CCTTGCCAGACGATTTG-3′) GAPDH (Forward: 5′-GAAGGTGAAGGTCGGAGT-3′, Reverse: 5′-CATGGGTGGAATCATATTGGAA-3′).

### 2.9. Immunohistochemical Assays

Cryopreserved specimens were serially sectioned at a 4-*µ*m thickness, fixed in Bouin's solution for 10 min, and then blocked with calf serum (20 g/l) for 1 h. The sections were then incubated with primary antibodies (1 : 200 dilution) against NLRP3 and IL-1*β* at 4°C overnight. After being thoroughly washed with phosphate-buffered saline (PBS), these tissue sections were incubated with HRP-conjugated secondary antibodies (1 : 100 dilution) for one hour at room temperature and then incubated with diaminobenzidine solution at 37°C for 15 min. Finally, the slides were redyed, dehydrated, and observed under a light microscope. Brown or brownish-yellow particles represented positive staining, and Image Pro Plus v6.0 software was used to analyze the images and determine the relative expression of the indicated proteins. Staining scores were calculated according to the percentage of coloured particles per field of view; positive staining less than 10% was considered a score of 0, 10–30% positive staining was considered a score of 1, 30–50% positive staining was considered a score of 2, 50–70% positive staining was considered a score of 3, and positive staining greater than 70% was considered a score of 4.

### 2.10. Quantification of New Blood Vessels and Fibroblasts

Tissue sections were fixed with neutral formaldehyde, dewaxed with xylene, washed with a graded ethanol series, and stained with HE. Each section was dehydrated again with a graded ethanol series, cleared with dimethylbenzene, and then observed under a microscope (×400). Five visual fields were randomly selected; for each field, the number of new blood vessels and the number of fibroblasts in the head and body of the pterygium were quantified. The average of the values from five visual fields was calculated for each slide.

### 2.11. Concentration Monitoring of MMC

After the last injection of MMC, 2 mL of venous blood was drawn from the patients. Concentration monitoring of MMC was carried out by switching HPLC (Bio-Rad, USA). Briefly, Econosphere C 18 (25 cm × 0.4 cm) was prepared as a chromatographic column, 20% acetonitrile phosphate buffer (pH = 8.0) was used as the mobile phase. The flow rate was adjusted to 3.0 mL/min, the switching time is 2.0 min, and the detection wavelength of UV is 363 nm. Blood samples were centrifuged for 5 min at 8 000 rpm/min, the supernatant was collected, and then blood concentration of MMC was detected. Less than 0.2 *μ*g/mL is considered to be below the instrument detection limit.

### 2.12. Statistical Analysis

Statistical analysis was performed using paired Student's *t*-test (two samples) and one-way ANOVA (multiple comparisons), *χ*^2^ tests (follow up of complications and pterygium recurrences after MMC injection) and was performed with SPSS 11.0 software. Spearman's correlation analysis was performed to evaluate the correlation between fibroblast numbers and NLRP3 expression. Repeated experiments were performed three times, and the results are expressed as the means ± SD; ^*∗*^*P* < 0.05 and ^*∗∗*^*P* < 0.01 were considered significant.

## 3. Results

### 3.1. No Serious Complications Result from Local Injection with a Low Dose of MMC

Upon follow-up, one of the patients in group II who received a local injection of MMC had binocular pain one day after the injection. However, this symptom can be alleviated by topical treatment with indomethacin eye drops for three days. The other patients had no complications, such as congestion, oedema, or ulcers, during the one-year follow-up. There were also no abnormal iris, sclera, cornea, intraocular pressure, lens, or fundus changes (Table 1). Concentration monitoring of MMC was also performed to evaluate the MMC toxicity; as demonstrated in Supplementary [Supplementary-material supplementary-material-1], MMC was hardly detected in the blood of group II patients. These findings indicate that a low-dose local injection of MMC does not only induce serious eye complications but also cause systemic adverse reactions.

### 3.2. Local Injection with a Low Dose of MMC Reduces the Postoperative Recurrence Rate of Pterygium

The patients with pterygium resection in groups I and II were in good condition, and there were no significant differences in surgical effects between these two groups (Table 1). In group II, upon follow-up observation, one case of substantial pterygium recurrence was found, three cases showed neovascularization extending towards the cornea, and the remaining patients showed a normal bulbar conjunctiva appearance. Accordingly, the overall recurrence rate was approximately 60% (Table 2). In contrast, in group I, which did not receive MMC injection, 9 cases of substantial pterygium recurrence and five cases of grade II recurrence were separately observed. In addition, hyperplasia of fibrous tissue, also classified as grade III recurrence, occurred in 6 cases. Based on our calculations, the overall recurrence rate in group I was approximately 44.4% (Table 2). These results suggest that MMC treatment through local low-dose injection can significantly decrease the recurrence rate of pterygium.

### 3.3. Local Injection with a Low Dose of MMC Suppresses Neovascularization and Fibroblast Proliferation in Pterygium Tissue

**Figure 1:**
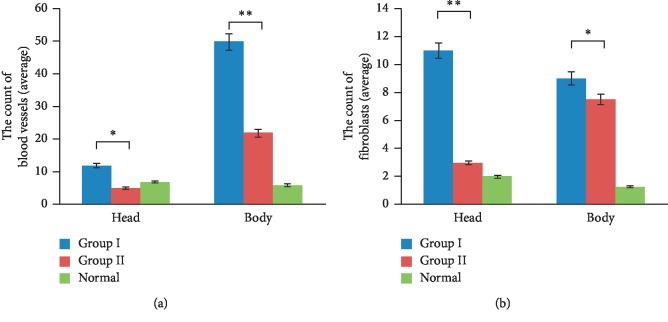
MMC inhibits corneal neovascularization and the proliferation of fibroblasts in pterygium tissues. (a, b) The number of blood vessels (a) and fibroblasts (b) in pterygium samples from group I and group II patients was counted under optical microscope after histochemical analysis (×400). Mean value of per field was shown (five randomly selected visual fields). Data are expressed as the means ± SD of three samples per group. ^*∗*^*P* < 0.05 and ^*∗∗*^*P* < 0.01. Normal corneal tissues serve as controls.

To further investigate the mechanism by which MMC prevents pterygium recurrence, we next evaluated the effect of MMC on neovascularization and fibroblast proliferation. As shown in Figure 1(a), the number of the blood vessels in the head and body of the pterygium specimens from group II patients was markedly lower than that in group I patients, and only a few group II specimens had scattered blood vessels. Furthermore, the number of fibroblasts in the head and body of the pterygium tissues obtained from group II patients were both lower than that in tissues from group I patients (Figure 1(b)). These findings reveal that MMC can inhibit neovascularization and fibroblast proliferation, thereby reducing the recurrence rate of pterygium; however, the potential mechanisms need further elucidation.

### 3.4. Local MMC Injection at a Low Dose Attenuates the Activation of the NLRP3/Caspase-1 Pathway in Pterygium Specimens

**Figure 2:**
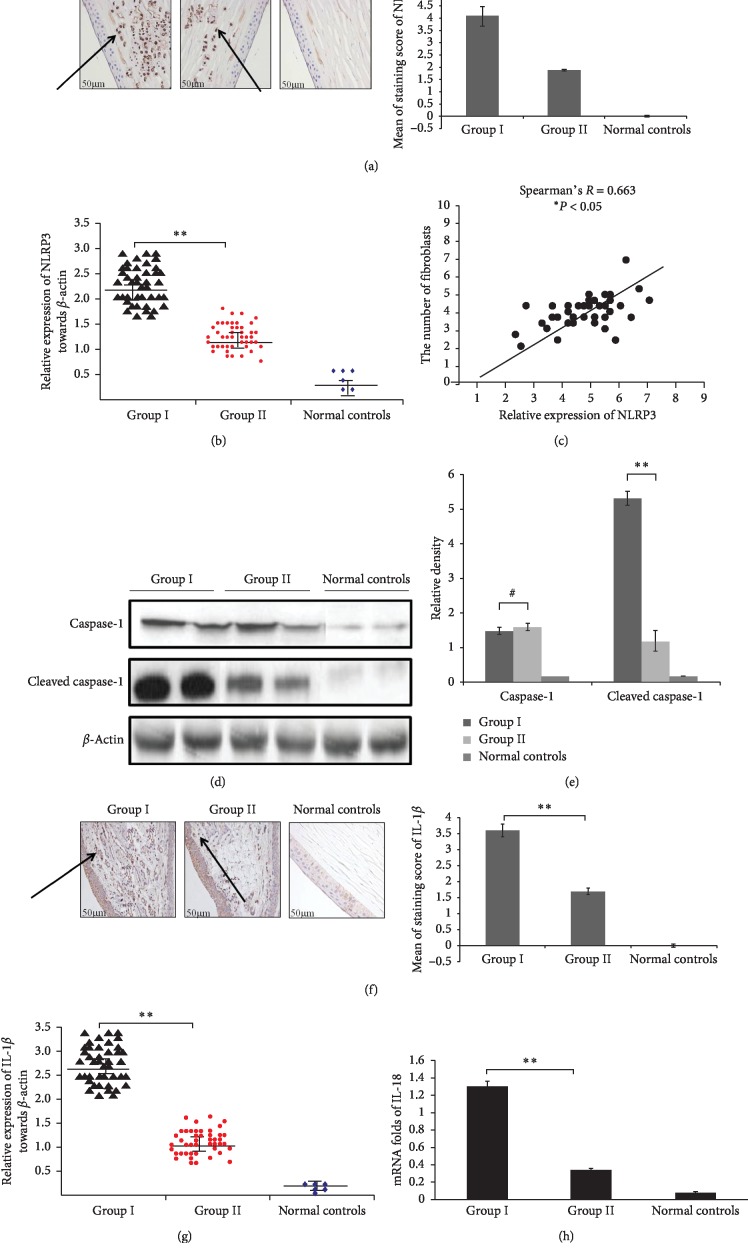
MMC suppresses the activation of NLRP3/Caspase-1 pathway in pterygium tissues. (a) NLRP3 expression in typical tissue sections from primary pterygium patients with or without MMC injection was determined by immuno-histochemistry analysis. Data were demonstrated as corresponding images (left panels) and staining score of NLRP3 with means ± SD (right panels). ^*∗∗*^*P* < 0.01. (b) Comparison of NLRP3 protein levels between group I and group II in primary pterygium patients was carried out by western blotting analysis. The expression levels of NLRP3 were normalized to those of *β*-actin, the horizontal line represents the median value, and the error bars indicate the SEM. ^*∗∗*^*P* < 0.01. (c) The positive relation between fibroblasts numbers and NLRP3 expression was determined by Spearman's correlation analysis. *β*-Actin protein was used as internal reference (Spearman's *R* = 0.663, ^*∗*^*P* < 0.05). (d, e) The protein expression of Caspase-1 and Cleaved Caspase-1 in group I and group II patients was determined by western blotting analysis. Data are shown as representatives (d) or relative densities compared with *β*-actin from three independent experiments with means ± SD (e) ^*∗∗*^*P* < 0.01 and ^#^*P* > 0.01. (f) The expression of IL-1*β* in primary pterygium patients from group I and group II was determined by immuno-histochemistry analysis. Data are represented as representatives (left panels) or means ± SD of score staining of IL-1*β* (right panels). ^*∗∗*^*P* < 0.01. (g) Relative gray values contrasted with *β*-actin assessed by western blotting analysis from three independent experiments with means ± SD. ^*∗∗*^*P* < 0.01. (h) The mRNA expression of IL-18 in pterygium tissues from group I and group II was measured by real-time PCR analysis. Data are represented as the relative expression towards GAPDH of means ± SD in three independent experiments. ^*∗∗*^*P* < 0.01. Normal corneal tissues were used as controls in all of above experiments.

To our knowledge, there are no reports on the association between the NLRP3 pathway and pterygium development or on the potential effect of MMC on the activation of the NLRP3 pathway in pterygium. Herein, *in situ* immunohistochemical assays detected visibly higher expression of NLRP3 in pterygium tissue from group II patients than in tissue from group I patients (Figure 2(a)). This result was also verified by western blot assay (Figure 2(b)). Based on the results, we considered the possibility that MMC suppresses the activation of the NLRP3/Caspase-1 pathway to weaken neovascularization and fibroblast proliferation in pterygium. Consistent with this possibility, the expression of NLRP3 was positively correlated with the number of fibroblasts (Figure 2(c)). Moreover, expression of Caspase-1 which represented an effector protein of NLRP3 inflammatory corpuscles was also determined, as depicted in Figures 2(d) and 2(e), MMC did not affect the protein level of Caspase-1 but markedly decreased the expression of Cleaved Caspase-1 in pterygium. As the important downstream molecules in the activated NLRP3/Caspase-1 pathway, IL-1*β* and IL-18, especially their cleaved forms, play a key role in the NLRP3-mediated inflammatory response. Further observation in our study revealed that MMC clearly decreased the protein levels of IL-1*β* (Figures 2(f) and 2(g)) in the pterygium specimens. In addition, the mRNA levels of IL-18 in pterygium tissues from group II were also notably lower than those in tissues from group I (Figure 2(h)). Therefore, MMC injection can attenuate the activation of the NLRP3/Caspase-1 pathway, thus inhibiting fibroblast proliferation and vascular hyperplasia and reducing the rate of pterygium recurrence.

### 3.5. Low-Dose Injection of MMC Downregulates the Expression of Inflammatory Factors in Pterygium Tissues

**Figure 3:**
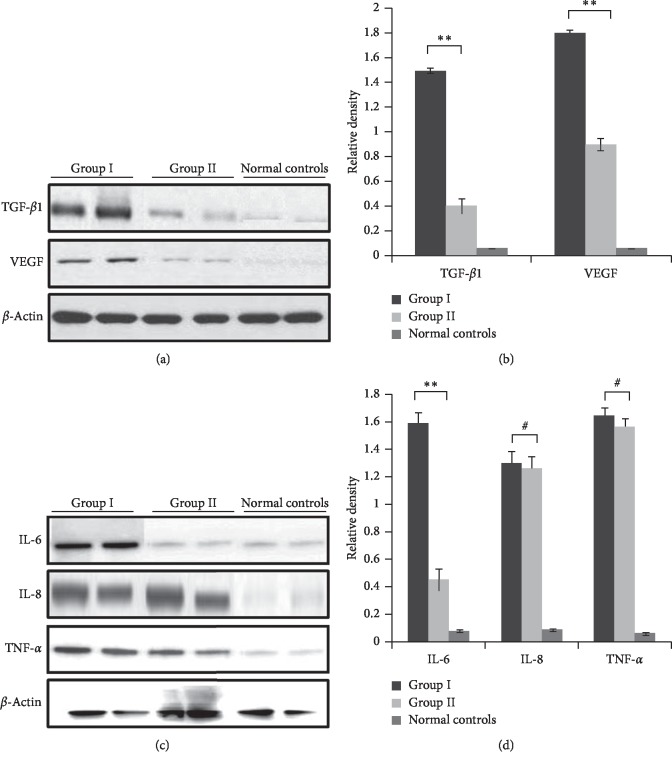
MMC decreases the expression of pro-inflammatory cytokines in pterygium tissues. (a, b) Expression of TGF-*β*1 and VEGF proteins in pterygium tissues from group I and group II patients was determined via western blotting analysis. (a) Data are also shown as representative of three independent experiments (b) ^*∗∗*^*P* < 0.01. (c, d) Expression of inflammatory factors such as IL-6, IL-8, and TNF-*α* was evaluated via western blotting analysis. Data are shown as representatives (c) and also are shown as representative of three independent experiments (d) ^*∗∗*^*P* < 0.01 and ^#^*P* > 0.05. Normal corneal tissues were used as controls.

Next, the effect of MMC injection on the expression of inflammatory factors including TGF-*β*1, VEGF, IL-6, IL-8, and TNF-*α* that have been reported to be highly expressed in pterygium tissues were also evaluated. As previously described, the expression of these inflammatory factors was minimal in normal conjunctival tissues but was significantly elevated in pterygium tissues (Figure 3), Moreover, the protein levels of TGF-*β*1 and VEGF in pterygium tissues from group II after MMC injection were significantly lower than those in tissues from group I (Figures 3(a) and 3(b)). MMC injection also significantly downregulated the expression of IL-6 in pterygium tissue from group II but had no effect on the protein levels of IL-8 and TNF-*α* (Figures 3(c) and 3(d)).

These results suggest that MMC inhibits fibroblast proliferation and neovascularization by downregulating the expression of TGF-*β*1, VEGF, and IL-6, thereby reducing the recurrence rate of pterygium.

## 4. Discussion

Pterygium is considered to be a chronic inflammation occurring in the limbal conjunctival vessels or conjunctival epithelium and is thought to result primarily from ultraviolet light-induced damage [16, 17]. Until now, various opinions regarding the mechanism of pterygium development have been held. The high expression of proliferating cell nuclear antigen (PCNA), Ki-67, P53, and matrix metalloproteinases in pterygium tissue indicates that pterygium is a tumour-like growth disorder [18]. Likewise, massive amounts of immunoglobulins, such as IgG and IgE, have been found in the basement membrane of primary and recurrent pterygium, suggesting that the pathogenesis of pterygium may be related to type I and type III allergies [19]. However, according to the clinical manifestations of pterygium development, including tissue degeneration, hypertrophy, hyperplasia, and congestion, it is generally believed that the interaction of immunocytes and inflammatory factors leads to fibroblast proliferation and neovascularization and induces the progression and recurrence of pterygium [1, 20]. Thus, elucidating the upstream mechanisms that regulate fibroblast proliferation and neovascularization, and on this basis, to find a way to prevent pterygium recurrence has become an emergent issue.

Recently, much valuable works have also suggested the importance of the NLRP3 inflammasome in the onset and development of many inflammation-related diseases [11, 13, 14]. However, few reports have focused on the association between the NLRP3 inflammasome and pterygium.

In this study, we found that NLRP3 was minimally expressed in normal conjunctival tissues, and its signalling proteins Cleaved Caspase-1 and Caspase-1 were also slightly expressed, however, in pterygium, the NLRP3/Caspase-1 pathway was abnormally activated, accompanied by the aberrant expression of IL-18 and IL-1*β*. More strikingly, there is a positive correlation between NLRP3 and the number of fibroblasts, thus, for the first time, suggesting that the activation of NLRP3/Caspase-1 pathway may be a powerful driving force for promoting angiogenesis and fibroblast proliferation to induce the recurrence of pterygium.

As reportedly, although IL-18 and IL-1*β* are both active downstream molecules in the NLRP3/Caspase-1 pathway and have been identified to mediate the formation of plasma membrane pores and promotes the release of inflammatory mediators in large quantities, resulting in the osmotic destruction of cells [21], the expression of IL-18 and IL-1*β* in NLRP3/Caspase-1 pathway is mutually antagonistic, and their biological functions differ greatly [22]. IL-18 is believed to be directly involved in anti-infection and antitumour immunity and exerts negative feedback on IL-1*β* expression; on the other hand, IL-1*β* mainly induces inflammation injury by promoting the secretion of IFN-*γ*, IL-2 and GM-CSF by NK cells and T cells [23]. However, as demonstrated in our study, it is surprising that both of IL-18 and IL-1*β* were aberrantly expressed concurrently after the NLRP3/Caspase-1 pathway was activated in pterygium (Figure 2), suggesting that IL-18 may not negatively regulate the expression of IL-1*β* when pterygium is present; however, the specific mechanism needs further study.

Emerging evidence has infirmed that infiltration of TGF-*β*1, VEGF, and IL-6 can promote fibroblast proliferation and vascularization that are involved in the occurrence and development of pterygium [8, 9, 24–26]. Therefore, it is reasonable to speculate that the perverse expression of these inflammatory factors may also contribute to the recurrence of pterygium. Herein, our findings confirm the above hypothesis that TGF-*β*1, VEGF, and IL-6 play promote pterygium occurrence, in part by inducing fibroblast proliferation and angiogenesis. The abovementioned results also confirm that inhibition of the expression of cytokines by means of small interfering RNA or antibody blockade can reduce the recurrence rate of pterygium, and we can go this further step.

Early studies have shown that MMC can inhibit the progression of fibroblasts from the G1 to the S phase and can thus impede the proliferation of subconjunctival fibroblasts [27]. Therefore, MMC has been gradually used as an auxiliary treatment for pterygium; however, MCC is a nonspecific antitumour drug, so high concentrations can, after alleviating the development of pterygium, cause serious side effects such as secondary glaucoma, iritis, corneal perforation, and mature cataracts [28, 29]. How to use mitomycin safely to prevent the of pterygium recurrence and avoid serious toxic and side effects has become an urgent problem to be solved.

In the present study, our results indicated that local administration of MMC at a low dose (0.4 mg/ml) clearly reduced the recurrence rate of pterygium, and no serious complications were observed in follow-up. In addition, preoperative injection of MMC can reduce the treatment dose and frequency, allowing patients to use the drug more easily and safely.

More importantly, our findings reveal that MMC reduces the recurrence rate of pterygium by suppressing angiogenesis and fibroblast proliferation via inhibiting NLRP3/Caspase-1 pathway activation and the expression of inflammatory factors such as TGF-*β*1, VEGF, IL-6. As recognized, the expression of TGF-*β*1, VEGF, and IL-6 is mainly regulated by TGF-*β*1/Smads, Wnt/beta-catenin, and Stat3 pathways, respectively [9, 30, 31]. So further studies are needed to elucidate the underlining mechanism through which MMC affects the expression of TGF-*β*1, VEGF, and IL-6.

Collectively, our findings identify that local injection of MMC at a low dose may be a potent candidate strategy for preventing the recurrence of pterygium after simple surgical resection.

## 5. Conclusion

Anyway, our study provides multiple potential targets, including NLRP3, TGF-*β*1, VEGF, IL-6, and IL-8, for reducing recurrence of pterygium. Moreover, to the best of our knowledge, this study is the first to demonstrate that local MMC injection at a low dose can not only inhibit the activation of the NLRP3/Caspase-1 pathway and the expression of IL-18 and IL-1*β* but also downregulate the expression of TGF-*β*1, VEGF, and IL-6, thus inhibiting fibroblast proliferation and neovascularization to effectively reduce the recurrence rate of pterygium. Most importantly, MMC may be an adjuvant therapy to prevent recurrences and provide a new strategy for the prophylactic treatment of pterygium.

## Figures and Tables

**Table 1:** Demographic and clinical data of pterygium patients in group I (no MMC injection) and group II (MMC injection).

	Group I (*n* = 45)	Group II (*n* = 50)	*P* value
Mean age (years)	35.45	47.32	0.733

Age range (years)	32–45	40–51	0.753

*Gender*			
Male	25 (55.5)	23 (46.0)	0.862
Female	20 (44.4)	22 (44.0)	0.863

*Laterality*			
Right	34 (75.5)	30 (60.0)	0.855
Left	11 (24.4)	20 (40.0)	0.466

Size of pterygium across limbus in length (mm)	3.23 ± 0.1 (1.075)	3.86 ± 0.2 (1.120)	0.625

*Observation on the effect operation resection*			
Tissue thinning	44 (97.7)	49 (98.0)	0.865
Vascular atrophy	45 (100.0)	50 (100.0)	0.896
Creeping of conjunctiva in operation eye	45 (100.0)	50 (100.0)	0.925

*Complications after MMC injection*			
Ophthalmic pain	—	2 (4.0)	0.002^*∗∗*^
Congestion and oedema	—	0 (0)	
Corneal ulcer	—	0 (0)	
Corneal colouring	—	0 (0)	
Lens opacities	—	0 (0)	

^*∗∗*^ *P*=0.002 was considered to be statistically significant (*P* < 0.05).

**Table 2:** Number of recurrences of pterygium patients in group I (no MMC injection) and group II (MMC injection).

Recurrent classification	No. of cases (group I)	No. of cases (group II)	*P* value
Normal conjunctival appearance (I)	25 (55.5)	47 (94.0)	0.007^*∗∗*^
Neovascularization in the cornea (II)	5 (11.1)	3 (6.0)	0.021^*∗*^
Fibrous hyperplasia (III)	6 (13.3)	0 (0)	0.009^*∗∗*^

^*∗*^ *P* < 0.05 and ^*∗∗*^*P* < 0.01.

## Data Availability

The datasets used and/or analyzed during the current study are available from the corresponding author on request.

## Supplementary Materials

Supplementary Materials Supplementary Figure 1: MMC was hardly detected in the blood of group II patients. Concentration monitoring of MMC in group II patients was carried out by switching HPLC, as described in Materials and Methods. Blood concentration of MMC in all of the group II patients was below the detection limit.
