# Supplementary material for: Low-Dose Mitomycin C Decreases the Postoperative Recurrence Rate of Pterygium by Perturbing NLRP3 Inflammatory Signalling Pathway and Suppressing the Expression of Inflammatory Factors
Source: J Ophthalmol. 2019 Nov 15;2019:9472782. doi: 10.1155/2019/9472782 (PMC6885197; doi:10.1155/2019/9472782)
Supplement: Supplementary Materials — Supplementary Figure 1: MMC was hardly detected in the blood of group II patients. Concentration monitoring of MMC in group II patients was carried out by switching HPLC, as described in Materials and Methods. Blood concentration of MMC in all of the group II patients was below the detection limit. [file 9472782.f1.pdf]

Supplementary Figure S1

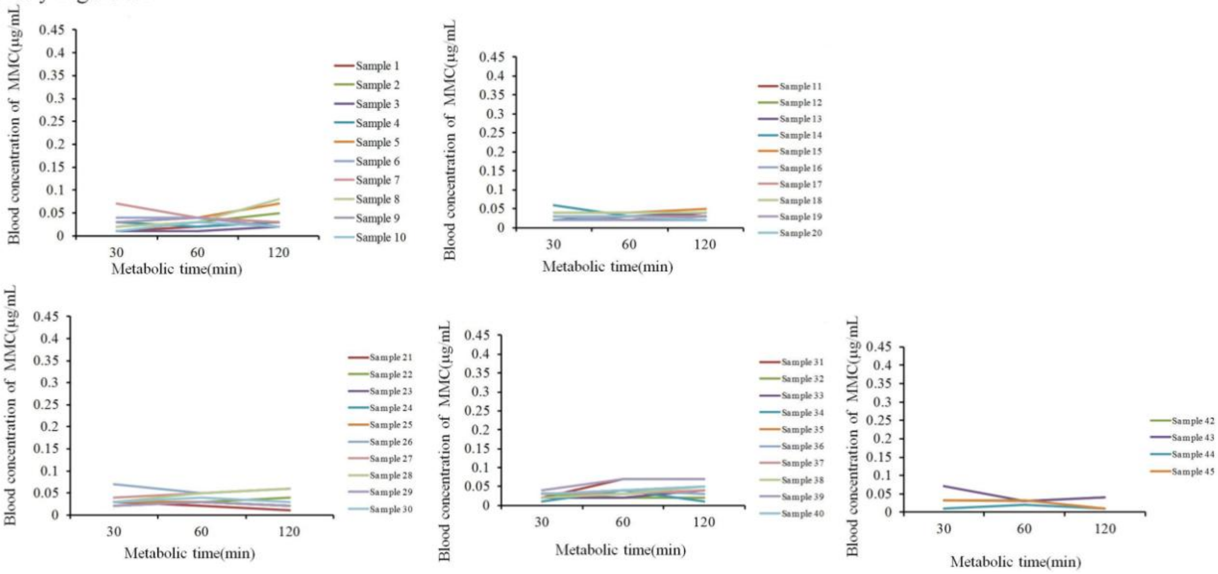

Supplementary Figure 1: MMC was hardly detected in the blood of Group II patients. Concentration Monitoring of MMC in Group II patients were carried out by switching HPLC, as described in the *Materials and Methods*. Blood concentration of MMC in all of the Group II patients were below the detection limit.
